# Initial Experience of Transthoracic Echocardiography at a Newly Operational Satellite Center in Hyderabad, Pakistan

**DOI:** 10.7759/cureus.5735

**Published:** 2019-09-23

**Authors:** Iram J Balouch, Nadeem Qamar, Lubna Baqai, Mariam Naz, Sumyia Gurmani, Musa Karim

**Affiliations:** 1 Echocardiography, National Institute of Cardiovascular Diseases, Hyderabad, PAK; 2 Cardiology, National Institute of Cardiovascular Diseases, Karachi, PAK; 3 Echocardiography, National Institute of Cardiovascular Diseases, Karachi, PAK; 4 Miscellaneous, National Institute of Cardiovascular Diseases, Karachi, PAK

**Keywords:** transthoracic, echocardiography, indication, initial experience, developing country

## Abstract

Introduction

Transthoracic echocardiography (TTE) is the primary noninvasive imaging modality for quantitative and qualitative evaluation of cardiac anatomy and function. The Hyderabad satellite center of National Institute of Cardiovascular Diseases (NICVD), Pakistan has recently started its operations, including TTE; therefore, it is imperative to assess the initial experience for the process improvement of the center. Therefore, the aim of this clinical audit was to review our initial experience of TTE at this newly operational satellite center.

Methods

In this clinical audit, we reviewed the records of patient undergone TTE at the echocardiography department of NICVD Hyderabad satellite center from May 2018 to October 2018. Demographic characteristics, clinical history, indications for the TTE, and echocardiographic diagnoses were reviewed.

Results

A total of 2,177 TTE procedures were performed during the study period of six months out of which 68.7% (1,496) were performed in male patients. Mean age of the patients was 50.83 ± 12.44 years with 48.2% (1,050) patients above 50 years of age. The most frequent indication for the procedure was cardiomyopathy, 54.1% (1,177), among other indications, native valve regurgitation was in 49.8% (1,085), ischemic heart disease in 23.2% (504), heart murmurs in 17.3% (377), cardiac masses in 14.3% (312), pericardial disease in 8.5% (184), pulmonary disease in 7.3% (160), infective endocarditis in 6.4% (139), aortic and major disease in 6.3% (138), and native valve stenosis in 5.4% (177) of the patients. Procedures were performed before coronary artery revascularization in 2.2% (47) and for prosthetic valve assessment in 1.7% (38) patients. The TTE was normal in 36.6% (796) patients, while the most frequent diagnosis was cardiomyopathy, 53.8% (1,172), among the other TTE findings valvular heart disease was in 21.8% (475), ischemic heart disease (IHD) in 21.6% (470), pericardial disease in 8.0% (175), and congenital heart disease (CHD) in 1.4% (30) patients.

Conclusion

This clinical audit showed the effective use of TTE as a noninvasive imaging modality for quantitative and qualitative evaluation of cardiac anatomy and function at a newly operational satellite center of a resource-limited country with normal TTE findings in only 36.6% of the patients.

## Introduction

The primary noninvasive imaging modality for the qualitative and quantitative evaluation of cardiac function and anatomy is transthoracic echocardiography (TTE) [1-2]. Two-dimensional (2D) echocardiography, pulsed and color Doppler, introduced in the 1970s, and 80s, introduced new techniques for the routine bedside assessment of cardiac hemodynamics and cardiac anatomy and provides tomographic imaging. Contrast echocardiography and Tissue Doppler have evolved as an important modality for the evaluation and assessment of blood flow and regional myocardial function [3]. Echocardiography is a versatile diagnostic modality, providing real-time structural and physiologic information with virtually no side effects [4]. However, in an increasingly cost-conscious health care environment, the expanding use of diagnostic imaging has come under scrutiny [5]. The aim of this paper is to report our initial experience at a newly operational satellite center with this procedure and to define the clinical cases seen in our setting.

The National Institute of Cardiovascular Diseases (NICVD) in collaboration with the Government of Sindh has formally inaugurated its third Satellite Centre at Hyderabad in Liaquat University of Medical and Health Sciences Hospital to provide comprehensive state-of-the-art cardiac care services to the people of Sindh at their doorstep. Services at the NICVD Satellite Centre at Hyderabad include cardiac emergency, primary percutaneous coronary intervention (PCI), echocardiography, adult and pediatric cardiology, and clinics. It is imperative to assess our initial experience for the process improvement of the center.

The aim of this study was to review our initial experience of TTE in terms of frequent indications for the procedure and main echocardiographic diagnoses among patients underwent TTE at the Hyderabad Satellite Center of the National Institute of Cardiovascular Diseases, Pakistan.

## Materials and methods

In this clinical audit, we reviewed the records of patient undergone TTE at the echocardiography department of the NICVD Hyderabad satellite center from May 2018 to October 2018. Consent was obtained from all the patients and data regarding demographic details, clinical history, and indications for the procedure were obtained using a structural proforma. Indications for the TTE were noted from the patient's referral note as cardiomyopathy, native valve regurgitation, ischemic heart disease (IHD), heart murmurs, cardiac masses, pericardial disease, pulmonary disease, infective endocarditis (IE), native valve stenosis, before coronary artery revascularization, prosthetic valve assessment, and aortic and major disease (aortic sclerosis, concomitant aortic stenosis, concomitant hypertrophic cardiomyopathy, myocarditis, or pericarditis, etc.). 

TTE procedures were performed by the cardiologist with a minimum of five years of working experience. TTE was performed using the commercially available echo-machine and a 2.5 to 5.0-µHz linear array transducer was performed on each subject in the partial decubitus position. Echocardiographic examination was performed in the parasternal long axis, short axis, two-chamber, apical four-chamber, five-chamber subcostal, and suprasternal views. Measurements and echocardiographic diagnoses were based on the standard (American Society of Echocardiography) criteria [6]. The final TTE diagnosis was noted which included normal TTE, cardiomyopathy, valvular heart disease, ischemic heart disease, pericardial disease, congenital heart disease, and others (segmental wall motion abnormality, etc.).

IBM SPSS Statistics for Windows, Version 21.0. (IBM Corp., Armonk, NY, US) was used for the analysis of data. Results were expressed as frequency (percentage) or mean ± standard deviation (SD). The indications and diagnosis of TTE were compared by gender and age groups by applying the chi-square test. Criteria for statistical significance were of *p*-value ≤0.05.

## Results

A total of 2,177 TTE procedures were performed during the study period of six months out of which 68.7% (1,496) were performed in male patients. The mean age of the patients was 50.83 ± 12.44 years with 48.2% (1,050) patients above 50 years of age. At baseline, 58.1% (1,264) were hypertensive, 46.4% (1,010) were diabetic, 56.5% (1,231) were dyslipidemic, 46.0% (1,001) were smokers, 44.1% (959) had family history of ischemic heart disease (IHD), and 6.6% (144) had chronic kidney disease (CKD). Baseline demographic and clinical characteristics are presented in Table 1.

**Table 1:** Baseline demographic and clinical characteristics SD, standard deviation

Characteristics	Total
N	2177
Gender	
Female	31.3% (681)
Male	68.7% (1496)
Age (years)	
Mean ± SD	50.83 ± 12.44
Up to 40 years	51.8% (1127)
> 40 years	48.2% (1050)
Risk factors	
Hypertension	58.1% (1264)
Diabetes mellitus	46.4% (1010)
Smoking	46% (1001)
Obesity	18.6% (406)
Dyslipidemia	56.5% (1231)
Family history of ischemic heart disease	44.1% (959)
Chronic kidney disease	6.6% (144)

The most frequent indication for the procedure was cardiomyopathy, 54.1% (1,177), among other indications, native valve regurgitation in 49.8% (1,085), ischemic heart disease in 23.2% (504), heart murmurs in 17.3% (377), cardiac masses in 14.3% (312), pericardial disease in 8.5% (184), pulmonary disease in 7.3% (160), infective endocarditis in 6.4% (139), aortic and major disease in 6.3% (138), and native valve stenosis in 5.4% (177). Procedures were performed before coronary artery revascularization in 2.2% (47) and for prosthetic valve assessment in 1.7% (38) patients. Indications and diagnosis of TTE by gender are presented in Table 2.

**Table 2:** Indications and diagnosis of TTE by gender TTE, transthoracic echocardiography

Characteristics	Gender		**p-value
	Female	Male	
N	681	1496	-
Indications for TTE			
Cardiomyopathy	249 (36.6%)	928 (62%)	<0.001*
Native valve regurgitation	302 (44.3%)	783 (52.3%)	<0.001*
Ischemic heart disease	152 (22.3%)	352 (23.5%)	0.535
Heart murmurs	139 (20.4%)	238 (15.9%)	0.01*
Cardiac masses	111 (16.3%)	201 (13.4%)	0.077
Pericardial disease	75 (11%)	109 (7.3%)	0.004*
Pulmonary disease	61 (9%)	99 (6.6%)	0.052
Infective endocarditis	63 (9.3%)	76 (5.1%)	<0.001*
Aortic and major disease	66 (9.7%)	72 (4.8%)	<0.001*
Native valve stenosis	54 (7.9%)	63 (4.2%)	<0.001*
Before coronary artery revascularization	23 (3.4%)	24 (1.6%)	0.008*
Prosthetic valve assessment	18 (2.6%)	20 (1.3%)	0.031*
Diagnosis of TTE			
Normal	336 (49.3%)	460 (30.7%)	<0.001*
Cardiomyopathy	251 (36.9%)	921 (61.6%)	<0.001*
Valvular heart disease	178 (26.1%)	297 (19.9%)	<0.001*
Ischemic heart disease	146 (21.4%)	324 (21.7%)	0.908
Pericardial disease	67 (9.8%)	108 (7.2%)	0.037*
Congenital heart disease	13 (1.9%)	17 (1.1%)	0.152
Others	6 (0.9%)	31 (2.1%)	0.046*

The TTE was normal in 36.6% (796) patients, while the most frequency diagnosis was cardiomyopathy, 53.8% (1,172), among the other TTE findings valvular heart disease in 21.8% (475), IHD in 21.6% (470), pericardial disease in 8.0% (175), and congenital heart disease (CHD) in 1.4% (30) patients. Indications and diagnosis of TTE by age are presented in Table 3.

**Table 3:** Indications and diagnosis of TTE by age TTE, transthoracic echocardiography

Characteristics	Age		**p-value
	Up to 40 Years	More than 40 Years	
N	1127	1050	-
Indications for TTE			
Cardiomyopathy	576 (51.1%)	601 (57.2%)	0.004*
Native valve regurgitation	519 (46.1%)	566 (53.9%)	<0.001*
Ischemic heart disease	272 (24.1%)	232 (22.1%)	0.260
Heart murmurs	199 (17.7%)	178 (17%)	0.664
Cardiac masses	175 (15.5%)	137 (13%)	0.099
Pericardial disease	93 (8.3%)	91 (8.7%)	0.728
Pulmonary disease	92 (8.2%)	68 (6.5%)	0.132
Infective endocarditis	87 (7.7%)	52 (5%)	0.008*
Aortic and major disease	74 (6.6%)	64 (6.1%)	0.652
Native valve stenosis	84 (7.5%)	33 (3.1%)	<0.001*
Before coronary artery revascularization	37 (3.3%)	10 (1%)	<0.001*
Prosthetic valve assessment	31 (2.8%)	7 (0.7%)	<0.001*
Diagnosis of TTE			
Normal	440 (39%)	356 (33.9%)	0.013*
Cardiomyopathy	575 (51%)	597 (56.9%)	0.006*
Valvular heart disease	222 (19.7%)	253 (24.1%)	0.013*
Ischemic heart disease	260 (23.1%)	210 (20%)	0.082
Pericardial disease	87 (7.7%)	88 (8.4%)	0.571
Congenital heart disease	25 (2.2%)	5 (0.5%)	<0.001*
Others	21 (1.9%)	16 (1.5%)	0.540

## Discussion

The purpose of this clinical audit study was to review our initial experience of TTE at this newly operational satellite center. A majority of patients were hypertensive; cardiomyopathy followed by native valve regurgitation were the frequent indications for TTE; TTE was normal in a significant number of patients, and consequently, cardiomyopathy followed by valvular heart disease were the most frequent diagnoses of TTE. Very limited audit reports of this nature are available for the South Asian region. The available data are predominantly of African origin.

Cardiomyopathy was found to be the most common heart disease in this study and was the diagnosis in 54.1% of the patients. A high prevalence of cardiomyopathy in our study is closer to the past study by Ghazni et al., which reported the prevalence of cardiomyopathy as high as 45.8% [7]. The reported prevalence of cardiomyopathy in the past studies varies from 9.7% to 45.8% depending on the geography of the population [7-10].

Among all the patients, 36.6% had a normal echocardiogram. This compares to 30.5% reported by Agomuoh et al. [11]. Our value for normal echocardiograms differs from that reported by Ike SO et al., 10.9%, because of the National Cardiothoracic Surgery Center at Enugu [12]. The reason for the difference is that their echocardiography service is less accessible; as expected, doctors will restrict the request to cases where it is vital to decision making and course of management.

With echocardiograms being much more accessible and hence affordable as in our center, its utility as a screening tool comes into place, and physicians may depend on it to help them screen out doubtful cases of structural heart disease. On this note, the higher frequency of normal echocardiograms we report would not necessarily be judged improper. This explanation is supported by the low frequency of normal echocardiograms in Ike SO's study; carried out at a time, 1991-2001, when echocardiography at Enugu served all the neighboring states [12].

Majority of our patients with echocardiographic diagnosis of valvular diseases were rheumatic in origin. This is understandable since the majority of the subjects were young (≤40 years) adult. Echocardiography has improved cardiac medicine in our center, especially with regard to the diagnosis of structural abnormalities. Some of our limitations include the non-availability of trans-esophageal echocardiography, and as such posterior cardiac structures cannot be more precisely studied. The lack of contrast echocardiography studies also implies we could not reliably exclude such conditions as the presence of intracardiac shunts. We also do not perform stress echocardiography which helps to exclude ischemic heart disease.

Our study suggests that timely management of ischemic heart disease and adequate control of hypertension should remain a clinical priority. Also, centers should pursue competence in full utilization of echocardiography, including stress echocardiography trans-esophageal echocardiography as well as endeavor to purchase machines that have full options.

## Conclusions

This study showed an effective use of TTE as a noninvasive imaging modality for quantitative and qualitative evaluation of cardiac anatomy and function at a newly operational satellite center of a resource-limited country with normal TTE findings in only 36.6% of the patients. The study showed that cardiomyopathy, valvular heart disease, IHD, pericardial disease, and congenital heart disease are the most frequent causes of heart disease at Satellite Centre Hyderabad, NICVD.
